# Avoidable readmission in Hong Kong - system, clinician, patient or social factor?

**DOI:** 10.1186/1472-6963-10-311

**Published:** 2010-11-17

**Authors:** Carrie HK Yam, Eliza LY Wong, Frank WK Chan, Michael CM Leung, Fiona YY Wong, Annie WL Cheung, EK Yeoh

**Affiliations:** 1 Division of Health System, Policy and Management, School of Public Health and Primary Care, The Chinese University of Hong Kong, Hong Kong

## Abstract

**Background:**

Studies that identify reasons for readmissions are gaining importance in the light of the changing demographics worldwide which has led to greater demand for hospital beds. It is essential to profile the prevalence of avoidable readmissions and understand its drivers so as to develop possible interventions for reducing readmissions that are preventable. The aim of this study is to identify the magnitude of avoidable readmissions, its contributing factors and costs in Hong Kong.

**Methods:**

This was a retrospective analysis of 332,453 inpatient admissions in the Medical specialty in public hospital system in Hong Kong in year 2007. A stratified random sample of patients with unplanned readmission within 30 days after discharge was selected for medical record reviews. Eight physicians reviewed patients' medical records and classified whether a readmission was avoidable according to an assessment checklist. The results were correlated with hospital inpatient data.

**Results:**

It was found that 40.8% of the 603 unplanned readmissions were judged avoidable by the reviewers. Avoidable readmissions were due to: clinician factor (42.3%) including low threshold for admission and premature discharge etc.; patient factor (including medical and health factor) (41.9%) such as relapse or progress of previous complaint, and compliance problems etc., followed by system factor (14.6%) including inadequate discharge planning, inadequate palliative care/terminal care, etc., and social factor (1.2%) such as carer system, lack of support and community services. After adjusting for patients' age, gender, principal diagnosis at previous discharge and readmission hospitals, the risk factors for avoidable readmissions in the total population i.e. all acute care admissions irrespective of whether there was a readmission or not, included patients with a longer length of stay, and with higher number of hospitalizations and attendance in public outpatient clinics and Accident and Emergency departments in the past 12 months. In the analysis of only unplanned readmissions, it was found that the concordance of the principal diagnosis for admission and readmission, and shorter time period between discharge and readmission were associated with avoidable readmissions.

**Conclusions:**

Our study found that almost half of the readmissions could have been prevented. They had been mainly due to clinician and patient factors, in particular, both of which were intimately related to clinical management and patient care. These readmissions could be prevented by a system of ongoing clinical review to examine the clinical practice/decision for discharge, and improving clinical care and enhancing patient knowledge of the early warning signs for relapse. The importance of adequate and appropriate ambulatory care to support the patients in the community was also a key finding to reduce avoidable readmissions. Education on patient self-management should also be enhanced to minimize the patient factors with regard to avoidable readmission. Our findings thus provide important insights into the development of an effective discharge planning system which should place patients and carers as the primacy focus of care by engaging them along with the healthcare professionals in the whole discharge planning process.

## Background

In an optimally functioning health care system, patients discharged from hospitals would obtain the needed and appropriate care in the community. In such a system, unplanned readmissions within a reasonably short period would in most instances be unavoidable. However, there have been studies which reported that readmissions to hospitals after discharge within a short duration are in fact often avoidable. The proportion of preventable readmissions among all readmissions ranges from 9 to 59% [1-6]. The reported readmission rates vary widely, depending on the patients selected for study, the duration of follow-up, whether the study is prospective or retrospective, the methodology and case-mix-related factors such as severity of illnesses [3,7]. Studies of avoidable readmissions have been rarely reported in Hong Kong. Two studies conducted in geriatric populations reported a preventable readmission rate of 7.7% among all first readmissions and 19% of the unplanned readmission within 28 days of hospital discharge [8,9].

Studies that identify reasons for readmissions are gaining importance in the light of the changing demographics worldwide. An aging population in a community generally leads to an increase in the burden of chronic illnesses, multiple morbidities and disability and the consequent demand for healthcare services, in particular for hospital beds. Due to the pressure on the demand for hospital beds, premature discharge of patients from acute hospitals has been observed [10]. One of the consequences of the early discharges is the subsequent high hospital readmission rate. In view of this, there is an increasing focus in identifying and reducing avoidable readmissions in order to reduce the demand for hospital beds and improve the quality of inpatient care.

How should one define a readmission as potentially avoidable? Some studies looked at it from system and clinician perspectives and defined potentially avoidable readmission as readmissions that could have been potentially avoided with better clinical management and stabilization prior to discharge, adequate outpatient care after discharge, appropriate discharge planning, and provision of resources at home which meet patient's needs [11]. Other studies examined "preventability" from the patient perspective: these reported the dietary and medication noncompliance of patients and inappropriateness/failure of the patient to seek prompt medical attention when symptoms recurred as factors contributing to avoidable readmissions [7]. To identify which readmissions are avoidable is in fact a daunting task. First, a clear definition of avoidable readmission is needed. Often, retrospective reviews of medical records based on professional assessments and judgments are required to identify the potentially avoidable cases. Conflicting views and conclusions may arise in the process, and further reviews and discussions are required until a consensus is reached on the assessment of avoidability. As health systems are organised and function differently, it is important to develop a consistent methodology/instrument with valid criteria which can be applied in a local context in determining which readmissions are avoidable. This will provide a consistent basis for studying avoidable readmissions in a hospital system and to enable valid assessments and comparisons within the system.

There have been a number of studies examining factors for readmission in different patient populations. In general, these factors can be grouped into four categories: patient, clinician, social and system factors. These factors are interrelated and each plays a role in preventing avoidable hospital readmissions. Patient factors which were associated with readmissions included socio-economic status, health status, and patient behaviours such as noncompliance with treatment [3,12]. Clinical factors refer to the adequacy and appropriateness of assessment and treatment such as inadequate workup of medical problem and suboptimal medical management/treatment [2,5,8,10]. Social factors included three aspects: coping, carer system and community service [12,13] and system factors refer to the availability, accessibility and coordination of care in the health care delivery system [2,5]. The studies of these risk factors for readmission, many of which are preventable, will provide a basis for developing programmes of intervention in reducing potentially avoidable readmission. Even the non-preventable factors such as some of the patient characteristics, namely age, socio-economic status and health condition may alert the system to design specific interventions to offset the risks associated with these factors.

Implementation of cost-effective services without affecting the quality of healthcare services adversely is an important issue worldwide; however, there are no simple solutions. It is important to identify the key factors relating to avoidable readmission and take such factors into account in formulating policy and planning practice changes. This study is the first attempt in Hong Kong to identify the magnitude of avoidable readmissions, its contributing factors and costs throughout the territory of this Special Administrative Region of China. The findings will provide the basis for assessment, planning, interventions and follow-up of patients to reduce avoidable readmissions and improve the quality of inpatient care.

## Methods

### Study setting

The health care system in Hong Kong is made up of both public and private sectors providing primary, secondary and tertiary care services. About 90% of hospital based health care is provided by the Hong Kong Hospital Authority which is responsible for all public hospitals. The Hospital Authority provides a comprehensive range of secondary and tertiary specialist care, medical and rehabilitation services to patients through 41 public hospitals, 48 specialist outpatient clinics, 74 general outpatient clinics, and a range of community outreach services that are organized into seven organisational clusters, each serving a geographical region with a population catchment of approximately 1 million people. The Government subsidises nearly 95% of the costs of the public outpatient and inpatient services through general taxation whereas the patients only need to pay 5% of the cost through user fees and charges out of their pocket [14].

There were 332,453 inpatient admissions between 1^st ^January 2007 and 31^st ^December 2007 in the Medical specialty of public hospitals managed by the Hong Kong Hospital Authority. In order to quantify the unplanned readmissions, the first or initial hospitalization in a series of hospitalization has to be identified first. The first hospitalization, so called index hospitalization, was identified as the first hospitalization appearing in the year of 2007. The second hospitalization was defined as a readmission with a predetermined timeframe. Each subsequent hospitalization e.g. the second admission then becomes an index admission to be compared with the next hospitalization. In our study, the 30-day unplanned readmission is defined as the readmission, which was not planned or prescheduled, to the same specialty through Accident & Emergency Department within 30 days to the index admission. The 30-day timeframe is commonly used in studies in the United States [2,5] whereas a 28-day timeframe is commonly used in the United Kingdom studies [1,12]. Based on statistical modelling such as survival analyses as well as sensitivity and specificity analyses, two studies mathematically demonstrated that 30-day was an optimal choice for identifying readmission [3,15]. Thus, we used 30-day timeframe as one of criteria to define the unplanned readmission.

### Study population

The study population was all the unplanned readmissions within 30 days in the Medical specialty of any acute public hospitals in Hong Kong between 1^st ^January 2007 and 31^st ^December 2007. There were 56,102 unplanned readmissions in 2007 (16.9% of all the 332,453 inpatient admissions in the Medical specialty). A retrospective analysis of a stratified random sample of medical records of patients with these unplanned readmissions was adopted. Based on an estimated rate of avoidable readmission at 15% and a desired confidence interval at 0.06 at 5% risk of error, the effective sample size was 550 by a Poisson's estimation model. We further assumed 10% of medical records as incomplete, thus 605 cases were randomly sampled from the 56,102 unplanned readmissions. A two-stage proportional stratified sampling was used i.e. firstly stratifying all unplanned readmissions by hospitals and by patients' age, and then a systematic sampling within each stratum.

### Assessment of avoidability of readmissions

To assess the avoidability of a readmission, an expert panel, which consisted of three clinical experts from the Medical specialty, was formed. A quality assessment checklist was developed to record the reasons for rehospitalization and the avoidability of readmission in terms of system, clinician, patient (or so-called medical and health factors) and social factors. These factors are selected according to the international published literature, which includes classification scheme for assessing readmissions [1], a categorization of the causes of readmission [3], a checklist for assessing preventability [5] and correlation of the principal and associated factors for readmission [12]. A panel of eight physicians used the checklist to classify the readmission as avoidable or unavoidable. All the members of expert panel and reviewers have at least 10 years of working experience in the profession and worked as grade of senior medical officer or above in the hospital. Each record was reviewed by two physicians independently. No reviewers reviewed the medical records from his or her cluster. They firstly recorded the reason for rehospitalization of a patient as relating to the following categories: (a) deterioration of existing disorder; (b) new medical conditions; (c) terminal care; (d) non-compliance with medication or diet; (e) unresolved medical problems; (f) complication of treatment other than drugs; (g) side effects of drugs/drug-drug interaction; (h) social problems; (i) psychological problems; and (j) others to give an overall impression of the readmission; and then identified one principal factor and any other possible factors contributing to readmission in terms of system, clinician, patient as well as social factors - where the factors were in more detailed classifications to assess the causes of readmissions. Then they determined the preventability of the hospitalization. The preventability of the readmission was based on the assessment of the principal factor as avoidable or not avoidable. If there was a difference between assessments among a pair of physicians, they were required to discuss the case together and come to an agreement. A consensus of opinion among the reviewers was required for a readmission to be classified as avoidable. If no agreement was reached, the case would then be submitted to the expert panel for a decision. To ascertain the reliability of the judgment, a random sample of 10% of subjects' records were assessed independently by members of the expert panel.

### Data sources

From the Hospital Authority inpatient database and the patients' medical records, the following information was also obtained: (a) socio-demographic data: age, sex, whether hospital fee paid by public assistance, and whether readmitted from elderly residential home; (b) clinical data: main principal diagnosis using International Statistical Classification of Disease and Related Health Problems (ICD) 9^th ^Revision and whether the principal diagnosis in both discharge and readmission episode was the same (based on first three digit of ICD 9^th ^code), length of stay, history of admission in the past 12 months, number of attendance to the public outpatient clinics including general outpatient and specialist outpatient clinics and Accident and Emergency Department in the past 12 months, number of drugs on discharge; (c) physical and cognitive function: mobility status, cognitive function, feeding problem, and instrumentation; (d) other discharge and readmission information: whether patient was transferred to rehabilitation during hospital stay, discharge destination, follow-up arrangement, time interval between discharge from index episode and readmission.

### Statistical analyses

The prevalence and contributing factors for avoidable readmissions were studied. The outcome measure of the study was whether the unplanned readmission was avoidable or not. To adjust for the clustering of patients within hospitals, a multilevel logistics regression was applied on all unplanned readmissions to examine the factors relating to the characteristics of index hospitalization that induced the avoidable readmission at 95% confidence intervals using the software Stata.

Another regression analysis on the total population at risk (all acute care hospitalizations which included: avoidable readmissions identified through medical record review in our study, unavoidable readmissions, and no readmissions) was further conducted to identify any variables in the total population at risk for readmissions which could provide alert for action before the event. The population studied represented a random sample of 3,642 hospitalizations between 1^st ^January and 31^st ^December 2007. The dependent variable corresponded to the count of avoidable readmission. The independent variables included age, sex, principal diagnosis of previous discharge, length of stay of previous linked episode, whether fee was paid by public assistance, number of attendance to public outpatient clinics and Accident & Emergency departments, and number of hospitalization in the past 12 months which were available in the inpatient database. A multilevel Poisson regression model was adopted to adjust for the clustering of patients within hospitals. Incidence rate ratios (IRR) were reported.

The maximum cost of avoidable readmission was estimated by multiplying the total number of bed-days for avoidable cases and the unit cost per acute inpatient-day.

### Ethics

Ethical approval was obtained for the study from the ethics committees in the Hong Kong Hospital Authority.

## Results

605 patients' medical records in 14 public hospitals were assessed by eight reviewers from October 2008 to March 2009. Two cases were found to be miscoded because they did not refer to a readmission. Instead, it was a transfer of patient between hospitals which had been wrongly coded as a readmission. The Kappa statistics measuring agreement between reviewers on the avoidability of readmission of these 603 cases was 0.5. There were 178 out of 603 cases (30%) that required discussion between pairs of reviewers to reach agreement and there was no discrepancy in the assessment of avoidability after discussion. The expert panel members randomly doubled checked 10% of the records and 4 out of 64 cases (6%) were revised by them due to different opinion on the principal factors and its preventability. There was reasonable agreement obtained between reviewers and in the double checking by the expert panel such that the accuracy of the estimate of avoidable readmissions could be assured.

### Patient characteristics

A total of 603 patients with a mean age of 74.8 years (SD 14.6 years) were studied; 53.2% were male (Table 1). They spent on average 9 days (SD 14 days) in hospital during the index admission with an average number of 7 drugs (SD 4 drugs) on discharge. Most of them were discharged home (65.8%) and had scheduled follow-up at public specialist outpatient clinics (69.0%), general outpatient clinics (8.1%) and other sub acute care or community services (17.4%). The majority of patients had normal cognitive function (70.6%), no feeding problem (84.2%), and did not require instrumentation (75.3%). 38.6% walked independently and 33.0% walked with support.

**Table 1:** Patient characteristics

	Not avoidable (n = 357)	Avoidable (n = 246)	All patients (N = 603)	P-value
**Socio-Demographics**				
Gender (Male) %	52.4	54.5	53.2	0.613
Age, in years	75.0	74.7	74.8	0.800
Admitted from elderly home %	34.7	30.1	32.8	0.232
Fee paid by public assistance %	54.3	60.6	56.9	0.129
				
**Clinical data**				
Main principal diagnosis *at readmission *%				
Symptoms, signs & ill-defined condition	16.5	13.8	15.4	0.099
Chronic obstructive pulmonary disease	12.6	11.8	12.3	
Pneumonia	13.7	7.7	11.3	
Heart failure	5.3	8.9	6.8	
Ischaemic heart disease	5.0	4.1	4.6	
Cancer	3.6	2.4	3.2	
Chronic renal failure	3.4	2.4	3.0	
Cerebrovascular diseases	2.2	1.6	2.0	
Diabetes	0.8	2.4	1.5	
Others	36.7	44.7	40.0	
				
Main principal diagnosis *at previous discharge *%				
Symptoms, signs & ill-defined condition	16.5	17.9	17.1	0.074
Chronic obstructive pulmonary disease	9.2	13.0	10.8	
Heart failure	8.7	8.5	8.6	
Pneumonia	10.1	5.7	8.3	
Chronic renal failure	3.1	5.3	4.0	
Ischaemic heart disease	2.8	3.3	3.0	
Cerebrovascular diseases	3.9	1.2	2.8	
Cancer	3.1	1.2	2.3	
Diabetes	1.1	2.8	1.8	
Others	41.5	41.1	41.3	
				
Same principal diagnosis in both discharge and readmission episode %	24.4	45.1	32.8	< 0.001
Length of stay in previous discharge, in days	9.9	8.5	9.3	0.217
No. of hospitalization in the past 12 months	4.1	4.5	4.3	0.290
No. of attendance to the public general & specialist outpatient clinics and Accident and Emergency Departments in the past 12 months	13.1	13.1	13.1	0.960
No. of medication on discharge	7.0	7.4	7.2	0.218

**Physical and cognitive function**				
Mobility status %				
Not available	0.3	0.0	0.2	0.493
Walk independent	36.7	41.5	38.6	
Walk with support	32.2	34.1	33.0	
Chairbound/Bedbound	30.5	24.4	28.1	
Others	0.3	0.0	0.2	
				
Cognitive function %				
Not available	1.7	2.0	1.8	0.127
Normal	66.9	76.0	70.6	
Dementia	19.9	12.6	16.9	
Impaired mental state	9.5	8.1	9.0	
Others	2.0	1.2	1.7	
				
Feeding problem %				
No problem	82.9	86.2	84.2	0.293
Enteral feeding	10.6	6.9	9.1	
Others	6.4	6.9	6.6	
				
Instrumentation %				
No	73.1	78.5	75.3	0.039
Foley Cath	5.3	4.9	5.1	
R/T	9.0	4.9	7.3	
PEG	0.0	0.4	0.2	
Tracheostomy	0.3	0.4	0.3	
CAPD	4.5	7.7	5.8	
Others	7.8	3.3	6.0	
				
**Other discharge and readmission information**				
Patient was transferred to Convalescence/Rehabilitation in previous discharge %	20.2	15.4	18.2	0.140
				
Discharge destination in previous discharge %				
Home	63.6	69.1	65.8	0.109
Residential home for elderly	36.1	29.7	33.5	
Others	0.3	1.2	0.7	
				
Follow-up arrangement in previous discharge %				
Specialist outpatient clinics	68.6	69.5	69.0	0.817
General outpatient clinics/Family Medicine clinics	7.3	9.3	8.1	0.361
Sub acute and community services	18.5	15.9	17.4	0.402
No follow-up	0.6	0.8	0.7	0.707
				
Time interval between discharge from index episode and readmission, in days	12.9	11.0	12.1	0.006

Note: P-value is used to show the significance of each independent variable with the outcome variable on whether the readmission was avoidable or not avoidable.

32.8% were readmitted from elderly residential home and 56.9% of patients paid the fee by public assistance. In the past 12 months, the patients had on average 4 hospitalizations and 13 public doctor consultations at public specialist and general outpatient clinics and Accident & Emergency Department. The principal diagnosis at readmission included: symptoms, signs and ill-defined condition (15.4%), followed by congestive obstructive pulmonary disease (12.3%), pneumonia (11.3%), heart failure (6.8%), ischaemic heart disease (4.6%), cancer (3.2%), chronic renal failure (3.0%), cerebrovascular diseases (2.0%) and diabetes (1.5%). Similar distributions of principal diagnosis were found in previous discharge that leads to the readmission. 32.8% of patients had the same principal diagnosis in the previous discharge and the readmission episode. On average, readmissions occurred 12 days (SD 8 days) after the previous index admission and 37.1% of readmissions occurred during the first week.

### Reasons for rehospitalization

The major reason for rehospitalization was deterioration of existing disorder (52.6%), followed by new medical conditions (43.9%), unresolved medical problems (15.3%), side effects of drugs/drug-drug interaction (6.1%), social problems (3.2%), non-compliance with medication or diet (3.0%), terminal care (2.7%), psychological problems (2.7%), complication of treatment other than drugs (1.7%) and others (1.2%) (Table 2). One principle factor was further identified to assess the principal cause of readmissions. The major principal factor contributing to unplanned readmission (n = 603) was found to be patient factor (74.0%), followed by clinician factor (19.4%), system factor (6.1%) and social factor (0.5%) (Table 3). The preventability of the readmission was based on an assessment of whether the principal factor could have been avoided.

**Table 2:** Reasons for rehospitalisation

Reasons	%
Deterioration of existing disorder	52.6
New medical conditions	43.9
Unresolved medical problems	15.3
Side effects of drugs/drug-drug interaction	6.1
Social problem	3.2
Non-compliance with medication or diet	3.0
Terminal care	2.7
Psychological problems	2.7
Complication of treatment other than drugs	1.7

Note: Multiple answers are allowed

**Table 3:** Principal factor contributing to readmission and its avoidability

	All patients (N = 603) %	Preventable cases (n = 246) %	% of preventability within each factor
**System factor**			
Inadequate discharge planning	2.3	5.7	100.0
Failure of postdischarge follow-up care	0.3	0.4	50.0
Lack of care coordination	0.3	0.8	100.0
Inadequate palliative care/terminal care	1.7	4.1	100.0
A need to transfer to Convalescence	1.3	3.3	100.0
Others	0.2	0.4	100.0
*Sub-total*	*6.1*	*14.6*	*97.3*
**Clinician factor**			
Premature discharge	4.0	9.3	95.8
Drug-related adverse event	4.5	7.7	70.4
Discharge with a missing/erroneous diagnosis/therapy	2.8	6.9	100.0
Suboptimal medical care	3.5	7.3	85.7
Threshold for admission	4.5	10.6	96.3
Others	0.2	0.4	100.0
*Sub-total*	*19.4*	*42.3*	*88.9*
**Patient factor**			
New complaint	32.3	3.3	4.1
Relapse of previous complaint	22.7	22.4	40.1
Progress of previous complaint	10.8	6.5	24.6
Recurrence of previous pathology	3.8	1.6	17.4
Compliance problem	4.1	8.1	80.0
Others	0.2	0.0	0.0
*Sub-total*	*74.0*	*41.9*	*23.1*
**Social factor**			
Patient coping	0.2	0.4	100.0
Carer system, lack of support & community services	0.3	0.8	100.0
Others	0.0	0.0	0.0
*Sub-total*	*0.5*	*1.2*	*100.0*
			
Total	100.0	100.0	40.8

### Avoidable readmission

246 (40.8%) of the 603 unplanned readmissions were considered avoidable by the reviewers. The estimated overall avoidable readmission rate was 6.9% (40.8% of the 16.9% unplanned readmission rate). Avoidable readmissions (n = 246) were due to: clinician factor (42.3%) including low threshold for admission (10.6%), premature discharge (9.3%) and drug-related adverse event (7.7%) etc.; patient factor (41.9%) including medical factors such as relapse or progress of previous complaint (28.9%), and compliance problems (8.1%), etc., followed by system factor (14.6%) including inadequate discharge planning (5.7%), inadequate palliative care/terminal care (4.1%), etc. and social factor (1.2%) covering carer system, lack of support and community services (0.8%), etc (Table 3). Only in a few cases social factor (n = 3) was found to be the principal factor contributing to the readmission.

With regard to the avoidability of each factor, system factor and clinician factor were highly avoidable, with avoidability of 97.3% and 88.9% respectively. Only 23.1% of patient factor was found to be avoidable.

The characteristics of the index admission were used to predict the risk factors for avoidable readmissions among readmission episodes. Multilevel logistic regression for the patient population with unplanned readmissions only (Table 4) found that after adjusting for patients' age, gender and principal diagnosis in previous discharge, the concordance of the principal diagnosis for admission and readmission increased the probability of the readmission being avoidable (Odd Ratio: 3.41). Also, the shorter the time between discharge and readmission, the higher the probability of the readmission being avoidable (Odd Ratio: 0.97). Other characteristics of index hospitalization such as length of stay, whether the patients was transferred to rehabilitation hospital, and whether received sub acute care services after discharge were not predictive of the readmission being avoidable. Patients' previous inpatient or outpatient healthcare services utilization and whether the patients received public assistance were not associated with avoidable readmission.

**Table 4:** Multilevel logistics regression on factors for avoidable readmissions on all unplanned readmissions

Factor	OR	95.0% C.I.
Same principal diagnosis in both discharge and readmission episode	3.41***	*(2.20 - 5.30)*
No. of attendance to public outpatient clinics and A&E departments in the past 12 months	0.99	(0.96 - 1.01)
No. of hospitalization in the past 12 months	1.01	(0.96 - 1.07)
Use of sub acute & community services	1.03	(0.61 - 1.75)
Length of stay (in days) of previous linked episode that induce the readmission	0.99	(0.98 - 1.01)
Time (in days) elapsed between discharge from index episode and readmission	0.97**	*(0.95 - 0.99)*
Fee was not paid by public assistance	1.19	(0.81 - 1.74)
Transferred to rehabilitation hospital	0.79	(0.45 - 1.39)
No. of drugs taken on previous discharge	1.03	(0.97 - 1.09)

Notes:

(i) No. of hospital = 14; No. of patients within each hospital ranged from 19-66

(ii) Intra class correlation = 9.9% implying the correlation of patients within hospital is small

(iii) Regression adjusted for age, gender and principal diagnosis of previous discharge

(iv) ** p-value < 0.01, *** p-value < 0.001, OR refers to Odd Ratio, CI refers to Confidence Interval

With regard to the risk factors for avoidable readmissions in the total population i.e. all acute care admissions irrespective of whether there was a readmission or not, after controlling the same factors above, the multilevel Poisson regression (Table 5) showed that patients with a longer length of stay (IRR = 1.01), with higher number of hospitalizations (IRR = 1.08) and attendance in public outpatient clinics and Accident and Emergency departments (IRR = 1.02) in the past 12 months were more likely to have avoidable readmission.

**Table 5:** Multilevel Poisson regression on factors for avoidable readmissions on the total population at risk (all acute care hospitalizations which included: avoidable readmissions, unavoidable readmissions and no readmissions)

Factor	IRR	95.0% C.I.
No. of attendance to public outpatient clinics and A&E departments in the past 12 months	1.02*	*(1.01 - 1.03)*
No. of hospitalization in the past 12 months	1.08***	*(1.05 - 1.10)*
Length of stay (in days) of previous linked episode that induce the readmission	1.01*	*(1.00 - 1.02)*
Fee was not paid by public assistance	1.14	(0.87 - 1.49)

Notes:

(i) Regression adjusted for age, gender and principal diagnosis of previous discharge

(ii) * p-value < 0.05, ** p-value < 0.01, *** p-value < 0.001, IRR refers to incidence rate ratio, CI refers to Confidence Interval

The overall avoidable hospital readmission rate for female and male were 6.5% and 7.2% respectively. The avoidable readmission episode comprised 8.3% of the total bed-days (158 897 bed-days) in 2007. Of which, 57 183 bed-days (mean stay of 6 days) were for female and 101 714 bed-days (mean stay of 8 days) were for male. With a unit cost of US$423 per average acute inpatient day, the maximum estimated cost for avoidable readmission in Hong Kong in 2007 was US$67 millions.

## Discussion

In the present study, we have quantified the magnitude of avoidable readmissions in an entire hospital system for a full calendar year and elucidated the factors contributing to the avoidable readmission. A high proportion of avoidable readmission (40.8%) was recorded in the present study compared to the two local previous studies conducted among geriatric populations in Hong Kong, probably due to the different population of patients studied, various definition or criteria used, and different methodologies [8,9]. Internationally, the proportion of all readmissions assessed to be preventable also varies greatly from 9-59% [1-6]. This implied a need for a consistent tool and methodology to measure the avoidable readmissions. Our study has designed a tool with reference to international papers which is tailored to the context of Hong Kong's health system. The tool was validated by the expert panels and reviewers to measure the rate of avoidable readmission. It serves as a baseline for future comparison and for monitoring purposes and to provide alerts for action for hospitals in Hong Kong. It can also serve as reference for other countries in using medical record review to assess the preventability of readmissions in the context of how the own health system is organised and functions.

Our study showed that readmission could have been prevented in almost half of the cases and had been mainly due to the clinician factor (42.3%) and patient factor (including medical and health factor) (41.9%), both of which were intimately related to clinical management and patient care. Our result is also consistent with studies of the nature of readmissions where relapse of medical problems predominate [4,12]. The findings supported that better clinical care and better communication between physicians and patients are required. The readmissions could have also been averted by enhancing patient knowledge of early warning signs for relapse and patient education to improve patient adherence to prescribed treatment and management regimens. Many patients' relapse was assessed to be avoidable since patients could have been treated in the community instead of going to the hospital. Adequate and easily accessible ambulatory care should be provided to keep the patients in the community. Education on patient self-management should also be enhanced to minimize the patient factors with regard to avoidable readmission. There is also a need for a system of ongoing clinical review to examine the clinical practice/decision for discharge. A clinical audit system should be considered. Thus, the use of educational programmes tailored to patients' needs and appropriate clinical guidelines are important components in reducing avoidable readmission [8].

With regard to the preventability of each factor, system and clinician factors were found to be highly avoidable. This is consistent with another study in UK on preventable readmission that a high proportion of readmissions for diagnostic testing, social problems, or problems in the delivery of medical care were deemed avoidable [5]. In our study, a low threshold for admission, premature discharge and inadequate discharge planning accounted for 25.6% of avoidable readmissions. These types of readmissions could be prevented by improving clinical pathways and better gate-keeping at the Accident and Emergency Departments. In addition, reducing preventable readmissions would very likely require better discharge planning and a coordinated approach that links the medical and social components of services that meet patients' needs. The discharge process should be patient-oriented. Apart from this, discharge planning also requires a standardized and validated tool to assess patients' medical, physical, functional, social, psychological and financial needs of patients which could be used to assess the appropriate sub acute care services system/mechanism to support the patients and carers in the community.

**Figure 1:**
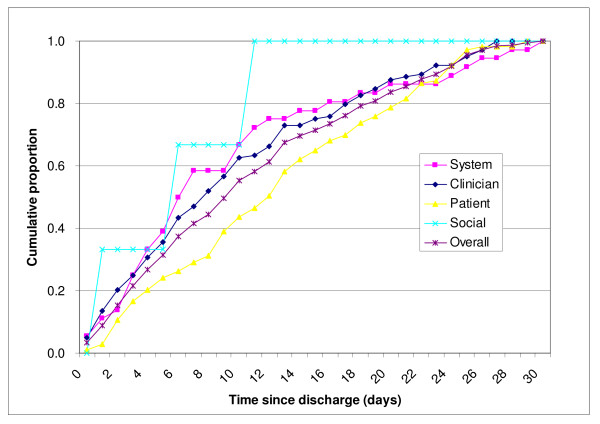
**Cumulative proportion of avoidable readmissions by principal factor (n = 246)**.

Avoidable readmission occurred earlier if it was related to system and clinician factors. The median avoidable readmission intervals between the index admission and readmission for system and clinician factors are 7 days and 8 days respectively compared with 10 days for all avoidable cases. The cumulative readmissions due to system and clinician factors also paralleled each other so closely that these factors must be considered to be interrelated (Figure 1). This affirmed the importance of enhancement in the system of care in order to reduce avoidable readmission.

Our results showed that socio-economic status of the patients was not correlated with avoidable readmission. We also found that if the diagnosis in the index admission and readmission was the same, the chance of the subsequent readmission to be avoidable was increased. In the analyse of the avoidability of hospitalization by disease category in previous discharge, it was found that there was a wide range of avoidability among the diseases, ranging from an avoidability rate of 28.0% for pneumonia to 49.2% for chronic obstructive pulmonary disease. The result provides insights for identifying which patient groups should be targeted for any intervention to reduce readmission. Priority for intervention should be targeted at these disease categories. It is also important to note that there were some hospitals with a high proportion of avoidable readmissions, while others had a relatively lower proportion (ranging from 15.8% - 64.4%). However, the number of cases sampled per hospital was quite small for some of the hospitals (from 19 patients to 66 patients per hospital) and it was not possible to draw conclusions on the geographic difference in avoidable readmissions in Hong Kong.

In the analysis of the total population at risk i.e. all acute care admissions, we found that patients with a longer stay in a previous discharge, higher number of hospitalizations and attendance in public outpatient clinic and Accident and Emergency departments in the past 12 months had a higher risk of avoidable readmissions. The findings were consistent with those reported in other studies [3,16]. The findings of these risk factors for avoidable readmissions permit the identification of groups of patients who are frequent users of health services for whom preventive measures could be implemented to reduce avoidable readmissions.

There are some important limitations in our study. We conducted a review of patients' medical records to examine the factors contributing to the avoidable readmissions. However, the hospital notes might not provide comprehensive information about the causes of readmission. A more detailed assessment involving patients and their relatives is required to provide a full picture on the reasons for avoidable readmissions. In addition, our results only represent patients from the Medical specialty. Patients from other specialties such as Surgery might have different contributing reasons for readmissions. Nevertheless, patients from Medical specialty yielded the highest rate of unplanned readmission in Hong Kong. With regard to the assessment process carried out by the reviewers, bias was avoided by not allowing the physicians to review records from his or her own cluster. However, since the medical records could not be taken away from the hospitals for privacy reasons, the physicians had to go to the respective hospitals to review the patients' records. Thus they knew the hospital and cluster where the patient was admitted, which may have induced some biases.

## Conclusions

Our study has characterized the drivers for avoidable hospital readmissions and quantified its burden. It is found that readmission could have been prevented in almost half of the cases and had been mainly due to clinician and patient factors, in particular, both of which were intimately related to clinical management and patient care. The avoidable readmissions could be prevented by improving clinical care and enhancing patient knowledge of the early warning signs for relapse. The importance of adequate and appropriate ambulatory care to support the patients in the community was also a key finding to reduce avoidable readmissions. Education on patient self-management should also be enhanced to minimize the patient factors with regard to avoidable readmission. The findings of this study have provided important insights into the development of an effective discharge planning system which should place patients and carers as the primacy focus of care by engaging them along with the healthcare professionals in the whole discharge planning process. This not only could safeguard against premature discharge and reduce the prevalence of avoidable readmissions, but also ensure quality of patient care by improving patient outcomes and enhancing their satisfaction.

## Competing interests

The authors declare that they have no competing interests.

## Authors' contributions

All authors carried out and designed the study. CHKY wrote the first draft of the manuscript and all authors made important contributions to the subsequent draft. All authors have seen and approved the final version.

## Pre-publication history

The pre-publication history for this paper can be accessed here:

http://www.biomedcentral.com/1472-6963/10/311/prepub
